# How to Evaluate Provincial Ecological Civilization Construction? The Case of Jiangsu Province, China

**DOI:** 10.3390/ijerph17155334

**Published:** 2020-07-24

**Authors:** Feng Dong, Yuling Pan, Xiaojie Zhang, Ziyuan Sun

**Affiliations:** School of Economics and Management, China University of Mining and Technology, Xuzhou 221116, China; ts19070137a31ld@cumt.edu.cn (Y.P.); ts18070017a3tm1@cumt.edu.cn (X.Z.)

**Keywords:** ecological civilization, spatio-temporal factor analysis, regional inequality

## Abstract

Ecological civilization is a transcendence of industrial civilization. The improvement of China’s ecological civilization system can not only provide developing countries with an empirical reference for ecological civilization construction, but also make a huge contribution to the progress of global ecological civilization. This paper focuses on improving the provincial ecological civilization evaluation system. First, the Provincial Ecological Civilization Construction Evaluation Index System is established according to the Green Development Index System issued by the Chinese government in 2016, and then the applicability of the Spatio-Temporal Factor Analysis (STFA) is verified as the evaluation method of the provincial ecological civilization construction level. Further, taking Jiangsu Province, China as an example, this paper evaluates the level of ecological civilization construction in each city of Jiangsu Province, and analyzes the main factors affecting ecological civilization based on the evaluation results. Finally, according to the relationship between the ecological civilization construction and economic quality of each city, the four-quadrant method is applied to divide Jiangsu Province into four types of regions to help each city position itself in ecological civilization construction. This paper mainly draws the following conclusions: (1) Due to its simple operation and accurate evaluation results, STFA is applicable in evaluating the provincial ecological civilization construction level. (2) Taking Jiangsu Province as an example, it is found that the level of ecological civilization construction in the southwestern region is higher than that of the northeast coastal region in Jiangsu. Three inequality indices are applied to measure the regional inequality of ecological civilization construction among 13 cities, which is relatively high in recent years. (3) By analyzing the results of STFA, it is found that economic quality and natural protection are not only the main factors affecting the ecological civilization construction in Jiangsu province, but also the main reasons for the regional heterogeneity of provincial ecological civilization. (4) For the four types of regions, suggestions are proposed according to the characteristics of each region to help improve the provincial ecological civilization construction level. This paper not only improves China’s ecological civilization construction system, but also provides information for the provincial ecological civilization construction in developing countries.

## 1. Introduction

In the last three hundred years, humans have gradually entered the stage of industrial civilization. In the first and middle stages of industrial civilization, many countries showed the industrial economic developing mode with high energy consumption and low efficiency, resulting in excessive emissions of pollutants and serious damage to the environment. Environmental problems have been mainly manifested in soil erosion, desertification, grassland degradation, water pollution and air pollution [1]. As a result, the characteristic of industrial civilization is that humans defeat nature, from land to sea and then to sky [2]. However, the ecological crisis in the last 50 years has conveyed that the earth can no longer support the continued development of industrial civilization [3]. In this context, ecological civilization emerges as the times require. If industrial civilization is a black civilization, then ecological civilization is a green civilization that carries the hope of sustainable development.

Environmental degradation is a common challenge facing humankind, and ecological civilization construction requires global participation [4]. As the largest developing country, China proposed an ecological civilization development mode to build a beautiful China in 2007 [5]. Ecological civilization is a concept proposed to encourage the sustainable development of resources and environment [6], the core issue of which is to properly deal with the relationship between human and nature, as well as the essential requirements of respect for nature, compliance with nature and protection of nature [7]. Ecological civilization involves such aspects as industrial structure, growth mode and consumption patterns, contrasting with the traditional economic growth mode of excessive consumption of resources and energy [8]. As a transcendence over industrial civilization, China proposed to build an environment-friendly and resource-saving society in the Eleventh Five-Year Plan [9]. In addition, China established Green Development as the main development direction in the Twelfth Five-Year Plan [10], and regarded it as the guiding ideology of the Thirteenth Five-Year Plan [11]. Since the 18th National Congress of the Communist Party, China has put forward a series of new concepts, new ideas and new strategies for the development of ecological civilization, emphasizing the development of a beautiful China and the construction of an ecological civilization system [12]. The development of China’s ecological civilization system not only provides developing countries with an empirical reference for ecological civilization construction, but also makes a huge contribution towards the progress of global ecological civilization.

Since the goal of ecological civilization development was put forward, all the provinces and cities of China have been actively responding to the call and vigorously promoting ecological civilization. However, the progress of ecological civilization differs among the provinces, because the ecological civilization indexes in various regions are quite different. Therefore, establishing demonstration provinces for ecological civilization construction is helpful in promoting ecological civilization [13]. To establish the demonstration provinces of ecological civilization, it is necessary to firstly determine the level of ecological civilization in various provinces, then identify the main factors influencing the level of ecological civilization, and finally implement the corresponding improvements. However, the evaluation method for the level of provincial ecological civilization construction is immature. In 2016, the Chinese government issued the Green Development Index System [14] for evaluating the level of national ecological civilization construction, while the evaluation system for provincial ecological civilization construction is blank, which not only affects the self-positioning of ecological civilization in China’s provinces, but also hinders the process of ecological civilization construction in China. Therefore, it is of great significance to put forward an evaluation system of ecological civilization construction at the provincial level.

In order to put forward the evaluation system of provincial ecological civilization construction, there are four issues needing to be addressed: (1) How should the evaluation indexes of provincial ecological civilization construction be selected? The selection of evaluation indexes is the first and most important step in the construction of the evaluation system, which determines the scientificity and practicability of the entire system. (2) How should the evaluation method be selected? The evaluation method should be accurate. In addition, the evaluation system is adopted by the government, which requires that the evaluation method is operable. (3) How to account for the evaluation results? The evaluation results not only convey the construction level of each city, but also can be analyzed to find the influencing factors of ecological civilization construction. (4) How to improve the construction of regional ecological civilization? The ultimate goal of the evaluation of ecological civilization construction is to improve the regional ecological civilization construction level, and the improvement paths should be explored according to differentiated regional characteristics.

**Figure 1:**
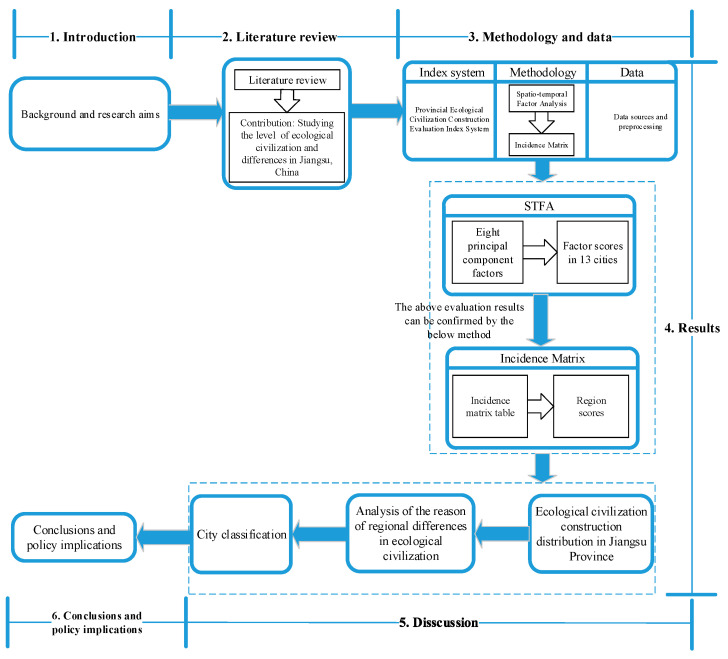
Framework of this study.

In order to solve the above problems, the rest of this paper is organized as follows. Section 2 is the literature review. Section 3 describes the research methods and data sources. Section 4 shows the results. Section 5 presents the discussion. The final part summarizes the research and puts forward some policy suggestions. The specific idea is shown in Figure 1.

## 2. Literature Review

Researchers worldwide have been studying the process of ecological civilization and its influencing factors for a long time. Based on coupling theory, some scholars have examined the coupling relationship among economic development, urbanization and ecological construction [15], and hold the opinion that the study of the interactive coupling effect between urbanization and ecological civilization will become the frontier field of international earth system science and sustainable science in the next 10 years. In addition, some scholars focus on the theory of the environmental Kuznet’s curve, as well as studying the correlation among carbon emission, population intensity and ecological construction [16]. They suggest that future ecological civilization development is inseparable from energy structure and urban structure. Other scholars regard resources, environment, ecology and society as an integrated system, and argue that the sustainable construction of ecology can guarantee the sustainable development of human society and nature [17,18,19].

To scientifically evaluate the level of ecological civilization, scholars have put forward diversified indexes of ecological civilization. Some indexes focus on the development of nature based on such subjects as ecological vitality, ecological environment and ecological security, and an evaluation index system for natural ecological civilization is established [20]. Some indexes focus on social development, such as economic growth [21], environmental carrying potential [22] and government policy support [23]. Dong and Liu [24] believe that policy support is an important force in solving environmental pollution and energy security issues, and the construction of ecological civilization is inseparable from policy support. Based on green industrial policy, green financial system and green financial policy, Lv and Qin [25] put forward a green economy system to help the construction of ecological civilization. In addition, scholars have explored the individual index in the index system in detail. Research on an individual index mainly focuses on the ‘direct interpretation’ indexes and ‘indirect interpretation’ indexes of ecological civilization construction. With regard to the direct interpretation indexes, regional pollution control and natural conservation are the key research problems [26]. For pollution control, scholars have addressed research problems from different perspectives, e.g., the rural air pollution control in Zhejiang Province based on anti-power consciousness [5], the problem of water quality pollution control in Jamaica Negril Ocean Park based on project transfer theory [27], and the emissions reduction of CO_2_ and PM_2.5_ based on the synergistic principle [28]. Some scholars explore the path of regional pollution control from the perspective of energy consumption [29] and industrial agglomeration [30]. For natural conservation, some scholars discuss the reasons for ecological decline in terms of the interaction between humans and nature [31]. Some scholars hold the opinion that the natural ecosystem is the basic link to ecological civilization and put forward an ecological compensation mechanism to protect forests, grasslands, wetlands, river basins, water resources, mineral resources and marine resources [32]. The research on indirect interpretation indexes mainly focuses on economic development, land management and infrastructure construction. Some scholars suggest that economy and ecological civilization are strongly correlated. By formulating quantitative indexes of green growth, practitioners can facilitate the transformation to a regional green economy, and achieve a ‘win-win’ outcome for the economy and ecology [33]. Li [34] proposes a cost–benefit function for the issue of balanced ecosystem development. The simulation result shows that the eco-economic system function can maintain a certain range of changes in each cycle and ensure the balanced development of the eco-economic system. Yao and Chang [35] consider that energy construction is related to energy economy development, and the development of energy construction is key to ecological civilization development. In the studies on land management related to ecological civilization, soil–water protection and arable land management are considered to be two basic elements of agro-ecological civilization system [36]. Accordingly, Qiao and Wang [37] put forward a three-phase agro-ecological civilization system. Infrastructure construction also has an impact on the development of ecological civilization. For example, agricultural infrastructure has a positive impact on the construction of an agro-ecosystem [38], and ecological construction and water resources management have a synergistic effect [39]. Therefore, taking ecosystem services into scale quantification projects and developing the regional green infrastructure can promote regional ecological health. Summarizing the research on various indexes can reveal two characteristics. First, at present, the development of ecological civilization no longer only consists of natural environmental elements, but further includes the development of social culture and a green economy. Second, ecological civilization is a systematic and long-term concept, and its influencing factors are interrelated and interactive.

According to the established index system, scholars have adopted various methods to measure ecological civilization construction level. Some scholars utilize indexes to evaluate ecological construction level [40], while other scholars analyze strengths, weaknesses, opportunities and threats to evaluate the influencing factors of urban ecological civilization development [41]. In addition, some scholars employ the decoupling index to measure the drivers of environmental change [42]. Ren and Toniolo [43] apply a life cycle sustainability assessment to comprehensively evaluate the development of regional environment, economy, energy, and industrial systems, and propose a decision-making method to achieve an optimal multi-system. Mahan and John [44] apply a decision support system based on Geographic Information System (GIS) to evaluate the ecological construction of the Dewa region with aquatic and terrestrial indexes. Lu and Zhang [45] perform the pressure-state–impact-response model and GIS model to study the ecological security of Changbai Mountain in China according to the area’s land use and forest coverage indexes. Regardless of the evaluation methods used, the key point lies in how to reasonably determine the weight of each index and conduct an evaluation from a balanced perspective. 

Both Spatio-Temporal Factor Analysis (STFA) and Incidence Matrix are the evaluation methods based on weighting. Therein, STFA is an objective method to determine weights by extracting common factors of variables through constructing panel data [46]. Different from factor analysis, STFA can analyze panel data and extract the spatial and temporal characteristics of each sample. Therefore, scholars extensively apply STFA in various fields, such as education evaluation [47], supply chain management [48] and food chain evaluation [16]. Compared with other evaluation methods, STFA is more suitable for panel data and multi-index evaluation system, which can classify indexes and extract the characteristics of classified indexes. This feature can simplify and extract the most valuable information of the evaluation object, so that the analysis can be performed in a targeted way [49]. Therefore, STFA is an evaluation method applicable to ecological civilization evaluation system, but it is difficult to guarantee the accuracy of the evaluation results. Incidence Matrix utilizes the matrix form to describe the relationship between the indexes and the evaluation object, which directly shows the influence of each index on the evaluation object [50]. If the evaluation results of the Incidence Matrix can verify the accuracy of the results of STFA, it is meaningful to use STFA as the evaluation method of the ecological civilization construction evaluation system.

By reviewing the present literature related to ecological civilization, some deficiencies in the existing studies are summarized as follows. First, insufficient consideration is given to the construction of evaluation index system, and there is an insufficient basis for selecting evaluation indexes. Second, the selection of the evaluation indexes is too subjective, and the readers cannot verify the correctness of the evaluation results. The weighting of the index system is also based on subjective weighting, which is not scientific. Third, the current studies on the ecological construction are mainly concerned with single area, neglecting the comparison analysis of different areas. It is necessary to measure the inequality of ecological civilization among regions and explore the reasons for the regional heterogeneity. Considering the deficiencies of the existing studies, this paper makes the following improvements. (1) Considering that the existing evaluation index system for ecological civilization construction is targeted at the national level, this paper constructs the Provincial Ecological Civilization Construction Evaluation Index System, which is applicable to the provincial ecological civilization construction evaluation. Therein, the indexes are selected according to the Green Development Index System issued by the Chinese government in December 2016. (2) For the evaluation index weighting and evaluation method, this paper utilizes the STFA method with objective weighting and Incidence Matrix with subjective weighting for comparison, thereby verifying the rationality of the evaluation results of STFA. (3) This study adopts three inequality indexes to measure the inequality of provincial ecological civilization construction, analyzes the influencing factors of regional ecological civilization construction, and explores the path of provincial ecological civilization development.

## 3. Methodology and Data

### 3.1. Index System Construction

**Figure 2:**
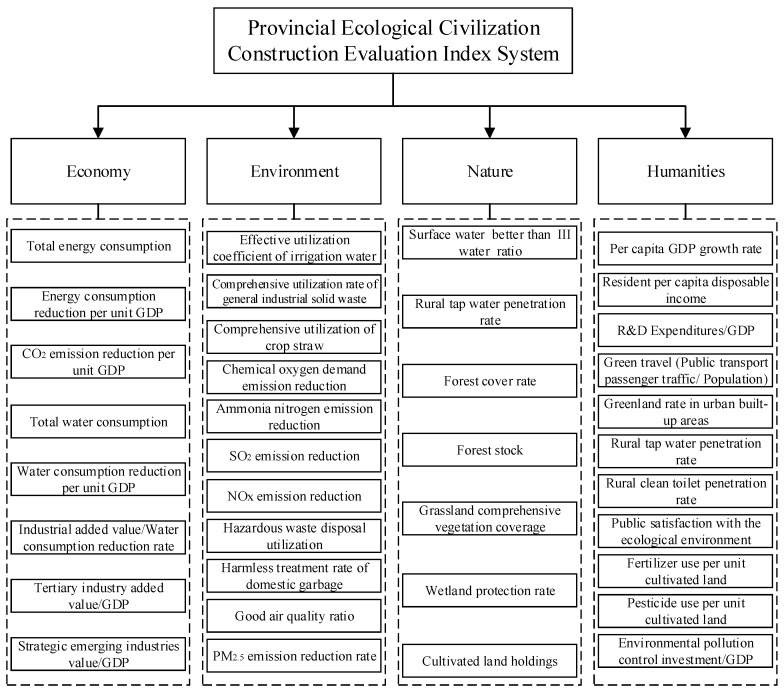
Provincial Ecological Civilization Construction Evaluation Index System.

The theme of this paper is “Ecological Civilization Construction”, and its core issue is to properly deal the relationship between human and nature. This paper considers two problems when constructing the evaluation index system of ecological civilization. First, the indexes should be diversified. Liu et al. [51] selected ecosystem services, ecological footprint and gross domestic product per capita to comprehensively evaluate the regional ecological civilization construction level. Zhang et al. [52] selected the degree of physiological equilibrium, the degree of psychological imbalance, the degree of imbalance of human and environment, the degree of human development, and the disharmony degree of economy and environment to explore the development trend of regional ecological civilization. The selection of these indexes starts from a micro perspective to discuss the regional ecological civilization. Similarly, this paper selects indexes from a macro perspective, including environmental indexes, natural indexes, economic indexes, and human–society indexes. Second, the index system is applicable at the provincial level. Each index takes into account the availability and comparability of data. In order to ensure the objectivity, rigorousness, scientificity and systematicness of the index system, this paper selects indexes from the Green Development Index System issued by the General Office of the State Council of China in 2016. However, the Green Development Index System is at the national level, and some of the indexes have no reference to the evaluation of provincial ecological civilization. For example, the area of marine protected areas is not applicable to non-coastal provinces; the growth rate of new energy vehicle ownership is not comparable, because some provinces have not yet launched new energy vehicle policies; the market share of green products is not comparable, because many regions do not publish relevant data, and so on. Based on the two considerations, this paper constructs a Provincial Ecological Civilization Construction Evaluation Index System with 37 indexes, as shown in Figure 2.

### 3.2. Evaluation Method

#### 3.2.1. STFA

STFA method involves six steps, which is operable and can be executed by Matlab.

The first step is to sort the original data into panel data, and confirm whether the panel data to be analyzed are suitable for STFA. In this paper, KMO and Bartlett’s sphericity test are used to judge whether the data in this paper are suitable for the STFA.

The second step is to normalize the panel data and obtain their correlation coefficient matrix.

The third step is to calculate the principal component score matrix, and its eigenvalue and eigenvector matrix. The common factors are extracted and the cumulative variance contribution rate of the factors is calculated.

The fourth step is factor rotation through maximum variance orthogonal rotation method.

The fifth step is to calculate each factor score extracted by STFA.

The sixth step is to calculate the score of the evaluation object. According to the score of each factor and its weight, this paper uses linear regression to calculate the final evaluation score.

#### 3.2.2. Incidence Matrix Analysis

Incidence Matrix involving four steps is taken to construct the incidence matrix, which can be executed by Stata.

The first step is the determination of the evaluation index system, which is shown in Figure 2.

The second step is the determination of the weight coefficient. The weight coefficients of the indexes in this paper refer to the Green Development Index System. The absent indexes’ weight of provincial evaluation index system compared to the Green Development Index System are evenly allocated to the indexes in the Provincial Ecological Civilization Construction Evaluation Index System.

The third step is to determine the score of individual indexes. The score of each index is based on the performance of the region in each year. This paper uses the value of each index after data preprocessing.

The fourth step is the calculation of the comprehensive evaluation value. According to the individual score and weight of each index, linear accumulation is performed to obtain the final evaluation score.

### 3.3. Data Sources and Preprocessing

#### 3.3.1. Data Sources

This paper takes Jiangsu Province as an example, and the samples are 13 cities under the jurisdiction of Jiangsu Province from 2012–2016. The data related to industrial production and daily life of public are derived from the statistical yearbooks of each city (e.g., fertilizer use per cultivated land, total water consumption, Tertiary industry added value/GDP, SO_2_ emissions reduction), the rest of the data come from the annual bulletins of the municipal environmental protection bureaus, water conservancy bureaus, forestry bureaus, and land resources bureaus (e.g., wetland protection rate, public satisfaction with the ecological environment, green travel).

#### 3.3.2. Data Preprocessing

The indexes in the evaluation index system are classified into positive indexes and negative indexes according to the evaluation function (positive index means that the growth of this index has a positive effect on the regional ecological civilization construction, and negative index means that the growth of this index has a negative effect on the regional ecological civilization construction), as well as into absolute indexes (absolute index represents the value of the current year, e.g., total water use, forest stock, wetland protection rate) and relative indexes (relative index shows the comparison value of the years before and after, e.g., SO_2_ emission reduction, PM_2.5_ emission reduction) according to the essence of the index. Therefore, each index needs to be dimensionless. It is first supposed to convert absolute indexes into relative indexes as Equation (1).

(1) $$x_{j}=\frac{X_{j}-X_{j-1}}{X_{j-1}}$$

Therein, *X_j_* is the value of a certain index in the year *j*, and *X_j_*_−1_ is the real value of a certain index in the year *j* − 1.

Then, the positive indexes and negative indexes need to be standardized, respectively, as Equations (2) and (3).

(2) $$\mathrm{Standardization}\;\mathrm{of}\;\mathrm{positive}\;\mathrm{indicators}:\;Z_{j}=\frac{x_{j}-x_{\min}}{x_{\max}-x_{\min}}$$

(3) $$\mathrm{Standardization}\;\mathrm{of}\;\mathrm{negative}\;\mathrm{indicators}:\;Z_{j}=\frac{x_{\max}-x_{j}}{x_{\max}-x_{\min}}$$

Therein, Z*_j_* is the standard value of the index *j*, *x_j_* is the real value of the index *j*, and *x*_max_ and *x*_min_ are the maximum and minimum values of the index *j*, respectively.

## 4. Results 

### 4.1. The Result of STFA

#### 4.1.1. STFA Pre-judgement

After sorting the original data into panel data, this paper first makes a pre-judgment on the panel data to determine whether it is suitable for STFA. The results are shown in Table 1. The KMO value is greater than 0.5, and the Bartlett sphericity test rejects the null hypothesis. They confirm that there is a correlation among the original variables, which is suitable for STFA.

#### 4.1.2. Extracting Common Factors

Based on the commonality of the variables, the factor variables are constructed. After the variance maximization orthogonal rotation, the top eight principal component factors are more than 1, and the factor cumulative variance contribution rate is 85.23% (as shown in Table 2), which is larger than 80%. Therefore, it is considered that the eight principal component factors conclude the main information from the panel data.

#### 4.1.3. Principal Component Factor Weight

**Figure 3:**
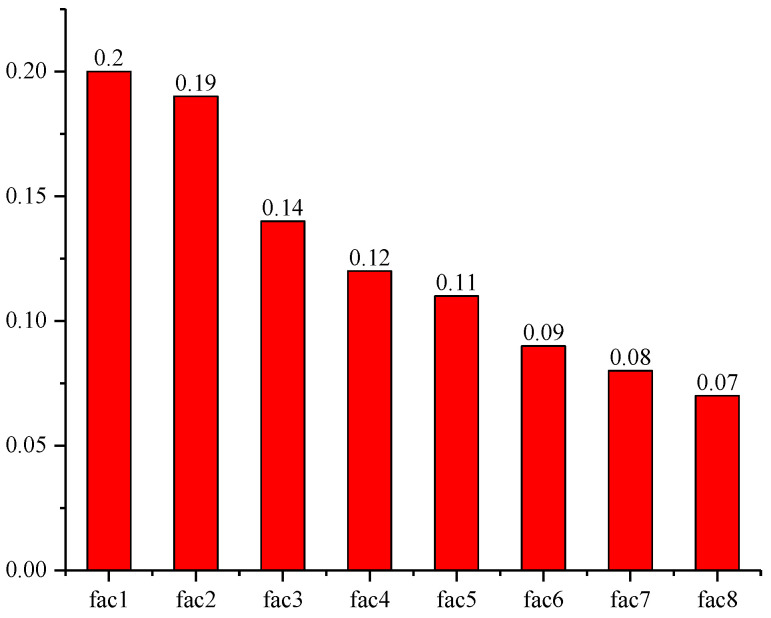
Factor weight distribution.

After determining the eight principal component factors, the contribution rate of each principal component factor to the cumulative contribution rate can be calculated as factor weight. The weights of the eight principal component factors are shown in Figure 3.

#### 4.1.4. Urban Ecological Civilization Score during 2012–2016

Multiplying the scores of each city on the principal component factors by the corresponding weights, the city’s annual evaluation scores can be obtained (as shown in Table 3), which indicates the annual ecological civilization level of the city.

#### 4.1.5. Hierarchical Structure of Principal Component Factor

According to the result of factor rotation, 37 indexes are assigned to eight principal component factors, and the eight principal components are named according to the index characteristics. The results are shown in Table 4. 

### 4.2. Evaluation Results of Incidence Matrix

According to the calculation method of the Incidence Matrix, the Incidence Matrix table is constructed, and the score of the ecological civilization construction level of each city is calculated, as shown in Table 5.

### 4.3. Results Comparison between STFA and Incidence Matrix

**Figure 4:**
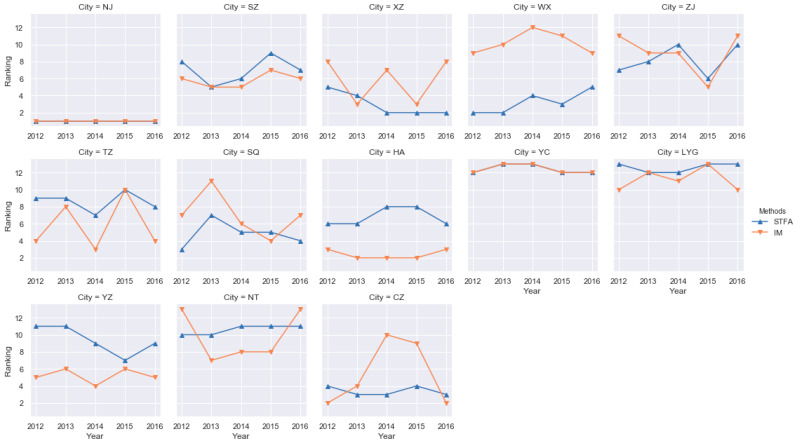
Comparison of the results through STFA and Incidence Matrix.

According to Table 3 and Table 5, the evaluation results of STFA and Incidence Matrix are numerically inconsistent, which is caused by the different data processing processes. In view of this, this paper uses ranking comparison to compare the two methods’ results. The comparison results are shown in the Figure 4.

It can be seen from Figure 4 that the evaluation results of STFA and Incidence Matrix are basically the same for 13 cities in Jiangsu Province from 2012 to 2016, which indicates that the STFA can accurately evaluate the ecological civilization construction in Jiangsu Province. The subsequent analysis is also based on the results of STFA.

## 5. Discussion

### 5.1. Ecological Civilization Construction Level in Jiangsu Province

Based on the evaluation results of STFA of each city in Jiangsu Province from 2012 to 2016, the average score of each city can be calculated to obtain the ecological civilization construction level in 13 cities. This paper uses the standard deviation method to grade the results of STFA. The specific results is shown in Table 6. Therein, the standard deviation method requires 0-1 normalization of the data to be classified.

**Figure 5:**
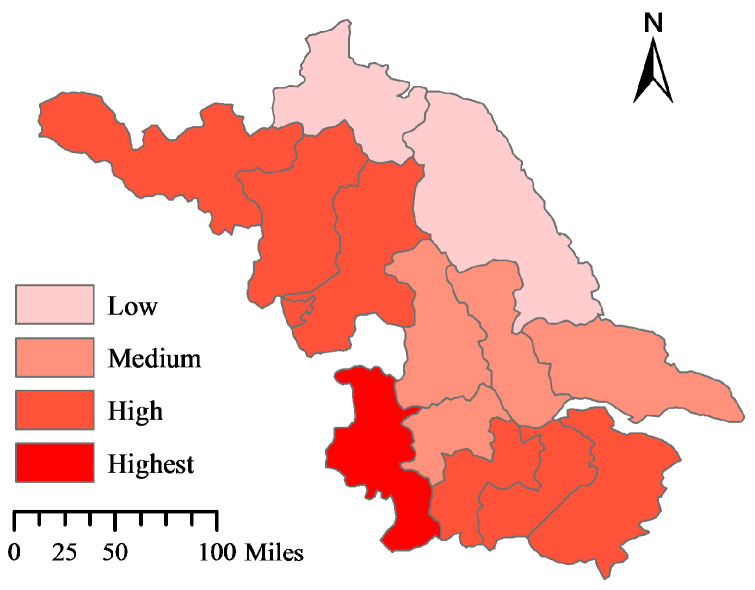
Ecological civilization development in Jiangsu Province.

The ecological civilization levels of the 13 cities from 2012 to 2016 follow the order: Nanjing > Xuzhou > Changzhou > Wuxi > Suqian > Huai’an > Suzhou > Zhenjiang > Taizhou > Yangzhou > Nantong > Yancheng > Lianyungang. The research of Liu et al. [53] shows that the level of ecological civilization construction in cities with good economic quality is relatively high. However, according to the empirical results, the economic level of Xuzhou is low, but the ecological civilization construction level is high, while the economic levels of Suzhou and Nantong are high, but the ecological civilization construction levels are low. Therefore, it is necessary to explore the diversified factors that affect the regional ecological civilization construction level. According to the result of standard deviation method, the 13 cities are divided into four types of area. The distribution is shown in Figure 5, which indicates that the level of ecological civilization in south-western Jiangsu is relatively higher than that in the north-eastern coastal areas.

**Figure 6:**
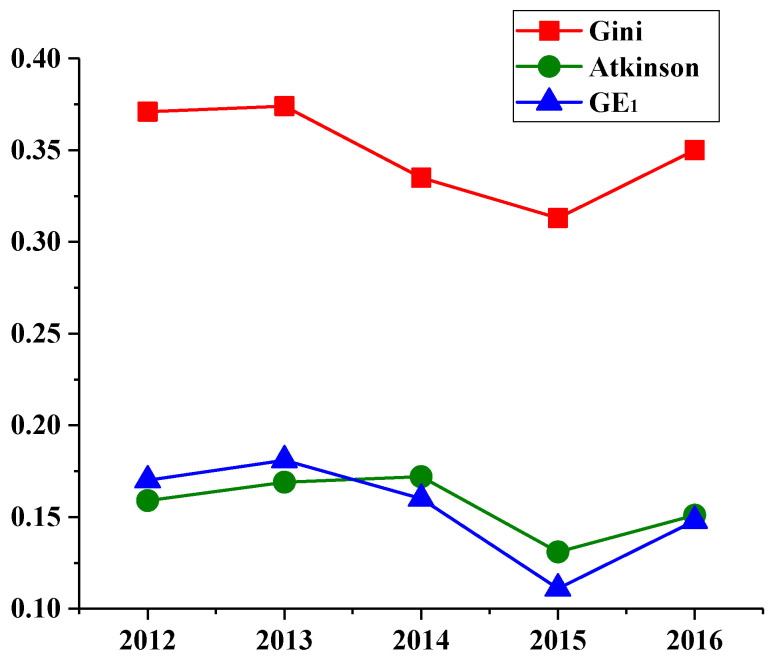
Inequality measurement of ecological civilization construction in Jiangsu Province.

The regional ecological civilization construction level in Jiangsu Province is quite different. This paper applies the inequality indexes to further explore the degree of inequality among cities in Jiangsu Province. In the field of inequality research, some scholars have adopted the Gini coefficient to investigate the differences in income distribution among residents. In addition, the Theil index (GE_1_) is applied to study the fairness of energy resource allocation [54], and the Atkinson index is also utilized to discuss the rationality of utility products [55]. According to the results of STFA, the three indexes (i.e., Gini coefficient, GE_1_ and Atkinson index) are utilized in this paper to measure the inequality of ecological civilization construction among 13 cities of Jiangsu Province during the period 2012–2016. The results are shown in Figure 6 and the bigger each index is, the larger the inequality is.

The inequality measurement is not only an important reference of the consistent degree of regional ecological civilization construction, but also analyzing the changing trend of regional ecological civilization construction over time. The average values of the Gini, GE_1_ and Atkinson index in 2012–2016 are 0.35, 0.16, and 0.15, respectively. From the perspective of relative inequality, the inequality of regional ecological civilization construction in Jiangsu Province has gradually decreased. However, from the perspective of absolute inequality, the inequality of regional ecological civilization construction in Jiangsu Province is still high, which indicates that the level of ecological civilization construction varies greatly among cities in Jiangsu Province.

### 5.2. Analysis of the Regional Heterogeneity of Ecological Civilization Construction

The ecological civilization construction shows great regional heterogeneity in Jiangsu Province. It is necessary to further analyze the reasons for such regional heterogeneity in ecological civilization construction. A systematic analysis on the regional disparities of the eight factors extracted from STFA will provide some evidence regarding the regional heterogeneity in ecological civilization construction.

**Figure 7:**
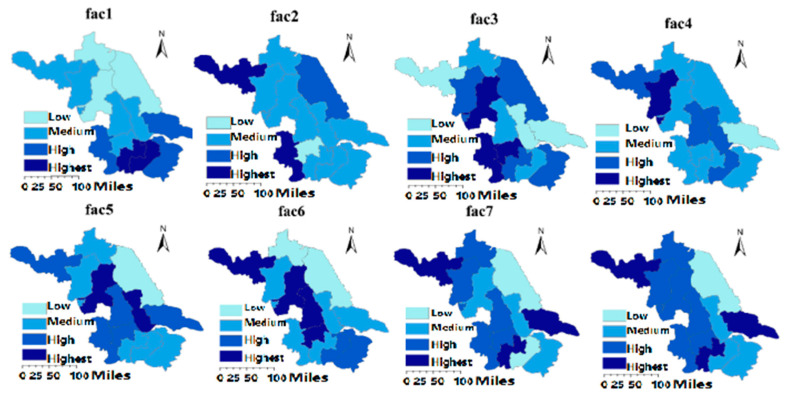
Spatial distribution of eight principal component factors.

As shown in Table 4, the eight principal component factors of ecological civilization construction include economic quality, natural protection, cultivated land quality, environmental quality, city quality, pollution control, rural construction and water quality. Figure 7 plots the geographic distribution of the eight factors in 13 cities in Jiangsu Province, and each factor is graded into four levels (see Table 6). It can be seen that each factor differs distinctly among 13 cities. 

(1) Economic quality in Jiangsu’s 13 cities from 2012 to 2016 follows the following order: (Wuxi, Changzhou) > (Nanjing, Suzhou, Nantong) > (Xuzhou, Suqian, Yangzhou, Taizhou, Zhenjiang) > (Huai’an, Lianyungang, Yancheng) (the cities in parentheses are at the same level, and the same below). The economic quality of the southern region is better than that of the northern region in Jiangsu Province in terms of spatial distribution, which indicates that the southern region is more economically developed than the northern region in Jiangsu Province. In addition, economic quality accounts for the largest proportion of regional ecological civilization construction evaluation. The difference in economic quality is the most important reason for the regional heterogeneity of ecological civilization construction;

(2) Natural protection, an important part of the ecological civilization construction, is the basis of social development. Natural protection reflects the natural environment protection and future sustainable development in a city. From 2012 to 2016, natural protection is presented in descending order as follows: (Nanjing, Xuzhou) > (Suqian, Yancheng) > (Suzhou, Wuxi, Taizhou, Lianyungang, Yangzhou, Nantong, Changzhou) > (Zhenjiang). In terms of regional distribution, the natural protection of the western region is better than that of eastern region in Jiangsu Province, which indicates that the western region places a higher emphasis on ecological protection, while natural sustainable development has not been considered reasonably in the eastern region;

(3) Cultivated land quality is a characteristic of green agriculture, which mainly includes two indexes: the amount of fertilizer and pesticide used per unit of cultivated land. Human survival depends on the cultivated land, and the quality of cultivated land reflects the attention paid to regional agricultural production. The cultivated land quality in descending order from 2012 to 2016 in 13 cities is as follows: (Nanjing, Zhenjiang, Huai’an) > (Suzhou, Suqian, Yancheng, Changzhou) > (Wuxi, Lianyungang, Yangzhou) > (Xuzhou, Taizhou, Nantong). In terms of cultivated land construction, the quality of cultivated land in the developed southwest region is higher than that in the developing northeast region. This result also shows that the dependence of economic development on agriculture is negatively correlated with cultivated land quality;

(4) Environmental quality represents the environmental factors closely related to the public concern, such as public satisfaction. The environmental quality from 2012 to 2016 in Jiangsu Province shows the following descending order: (Suqian) > (Xuzhou, Wuxi, Taizhou, Yangzhou) > (Nanjing, Suzhou, Zhenjiang, Huai’an, Yancheng, Lianyungang, Changzhou) > (Nantong). That is, environmental quality is relatively higher in the northwest and central Jiangsu, but lower in the southwest and eastern regions;

(5) Urban quality indicates the quality of urban development, including the status of urban industrial development and the quality of urban life. The urban quality from 2012 to 2016 in Jiangsu Province is shown in descending order as: (Taizhou, Huai’an) > (Nanjing, Xuzhou, Zhenjiang, Yangzhou, Nantong) > (Suzhou, Wuxi, Suqian, Lianyungang, Changzhou) > (Yancheng). The urban quality of southwestern Jiangsu is much better than that of northeastern Jiangsu, which implies that urban life quality in southwest Jiangsu is much better;

(6) Pollution control, including the treatments of waste gas, waste water, and air pollutants, reflects the attention paid to regional living environment. The pollution control from 2012 to 2016 in Jiangsu Province is exhibited in descending order as follows: (Xuzhou, Huai’an, Yangzhou) > (Suzhou, Wuxi, Taizhou) > (Nanjing, Suqian, Nantong, Changzhou) > (Yancheng, Lianyungang). The regional heterogeneity in pollution control indicates that the southwestern region of Jiangsu Province shows better performances on pollution control, while the northeast region shows relatively poor performance in pollution monitoring and treatment, which should be further strengthened;

(7) Rural construction, as an important part of ecological civilization construction, reflects the attention paid to rural ecological construction. The rural construction from 2012 to 2016 in Jiangsu Province is presented in the following descending order: (Xuzhou, Nantong, Changzhou) > (Nanjing, Zhenjiang, Suqian, Lianyungang, Yangzhou) > (Suzhou, Taizhou, Huai’an) > (Wuxi, Yancheng). It shows that rural construction in the western region of Jiangsu Province is better than that in the eastern region;

(8) Water quality reflects the protection of surface water. Different from domestic and industrial water, surface water serves as a means of regional storage of water resources, whose quality is critical for the sustainable development of future water resources. The water quality from 2012 to 2016 in Jiangsu Province is shown in descending order as: (Xuzhou, Changzhou) > (Taizhou, Huai’an, Yancheng) > (Nanjing, Suzhou, Wuxi, Zhenjiang, Suqian, Yangzhou, Nantong) > (Lianyungang). The spatial distribution shows that the water quality construction in the western region of Jiangsu Province is better than that in the eastern region.

According to the above analysis of the eight factors in Jiangsu’s 13 cities, it is easily found that the regional disparities of the eight factors have caused the regional heterogeneity of ecological civilization construction among 13 cities in Jiangsu Province. This conclusion is similar to the research by Huan [56], stating that economic development, urban development, energy structure and nature protection are the important factors affecting the ecological civilization construction. Compared with his research, this paper further finds that economic quality and natural protection have the greatest impact on the ecological civilization construction. The cities with the highest rankings for ecological civilization construction have higher economic quality and natural protection, while the lower ranking cities are the opposite. It indicates that the regional ecological civilization construction is closely related to the economic quality and natural protection. Other factors also have some impact on the achievements of ecological civilization; in essence, these factors are highly associated with economy and nature.

### 5.3. Exploring the Path of Ecological Civilization Construction 

**Figure 8:**
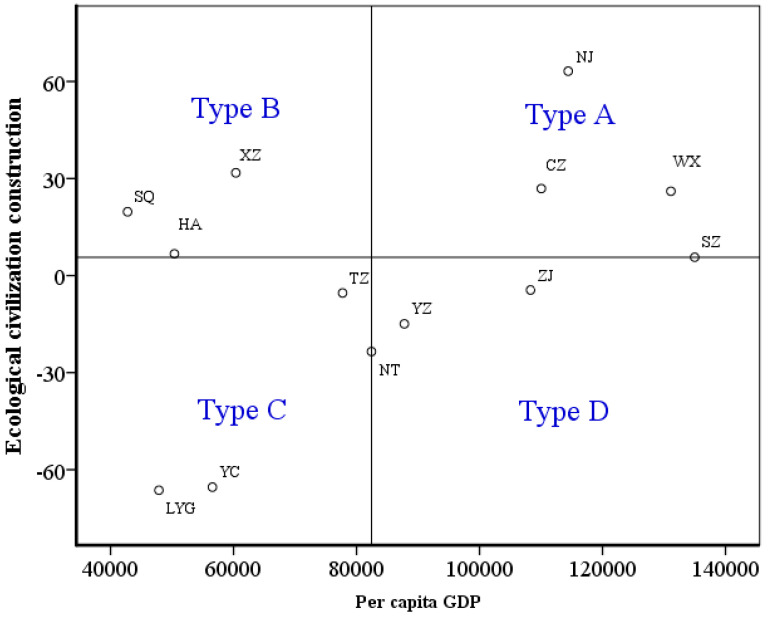
Relationship between ecological civilization level and per capita GDP in Jiangsu Province.

On the basis of the foregoing analysis, the relationship between the level of ecological civilization and the economic development is analyzed by constructing a four-quadrant graph (as shown in Figure 8). The vertical axis represents the level of ecological civilization, and the dividing standard of the vertical axis is the average value of ecological civilization construction score in the 13 cities in Jiangsu Province. The horizontal axis denotes per capita GDP, and the dividing standard of the horizontal axis is the average per capita GDP of the 13 cities in Jiangsu Province. 

In Figure 8, Type A includes Nanjing, Changzhouand Wuxi. These cities have relatively high ecological civilization and economic quality. On the basis of maintaining economic quality and natural protection, such cities are supposed to focus on investment in the indexes such as rural construction and pollution control, and emphasize the construction of ‘beautiful rural areas’ and ‘beautiful cities’.

Type B includes Xuzhou, Suqian and Huai’an, and the ecological civilization levels in such cities are relatively high, while the economic quality is poor. It is supposed to actively develop the secondary and tertiary industries, strengthen the environmental governance and monitoring, improve the property rights system of various natural resources assets, and promote the accountability and ecological civilization performance evaluation system.

Type C includes Lianyungang, Yancheng and Taizhou. The ecological civilization levels in such cities are low and the economic quality is poor. Such cities are near to coastal area. They are supposed to actively take coastal advantages, improve the industrial construction structure, transform the industrial structure, and pay attention to the development of coastal tourism. In addition, they can also open up trading ports to develop logistics and international trade, which will form a coastal industry and urban belt with other cities.

Type D includes Nantong, Yangzhou, Zhenjiang and Suzhou. The ecological civilization levels in such areas are relatively low, but the economy is relatively developed. The industrial structures of such cities mainly depend on the secondary industry, which consume a huge amount of energy. Therefore, they are supposed to focus on the development of the tertiary industry. While focusing on economic quality, they should also pay attention to the sustainable development of cultivated land and land protection, and avoid exceeding the land-carrying capacity. Besides, it is necessary to enhance environmental governance and pollution emission monitoring, which can help reform the ecological environment supervision system.

## 6. Conclusions and Policy Recommendations

The results from this paper support the following conclusions. 

(1) The provincial ecological civilization construction evaluation index system is established, and the STFA is proved to be feasible for evaluating provincial ecological civilization construction;

(2) According to the evaluation results of STFA, the level of ecological civilization construction in Jiangsu Province is high in the south-western region and low in the north-eastern coastal region;

(3) The ecological civilization construction varies greatly among cities in Jiangsu. The results of inequality measurement of ecological civilization construction show that the regional inequality has gradually decreased, but still remains high. Moreover, unequal development among cities leads to regional heterogeneity of ecological civilization in province;

(4) After analyzing the eight principal component factors, it is found that economic construction and natural protection have the largest weight, which have the greatest impact on regional ecological civilization construction. In addition, they are also the main reasons for the regional heterogeneity of ecological civilization construction;

(5) According to the results of ecological civilization-economic construction, 13 cities in Jiangsu Province are divided into four types of area, and this paper explores the improvement path of ecological civilization in Jiangsu Province. Therein, type A includes Nanjing, Changzhou and Wuxi, type B includes Xuzhou, Suqian and Huai’an, type C includes Lianyungang, Yancheng and Taizhou, and type D includes Nantong, Yangzhou and Zhenjiang.

Based on the above conclusions, this paper proposes the following policy recommendations:

The first task is to vigorously promote the construction of environmental quality. Regardless of the provincial or city level, the impact of environmental quality on regional ecological civilization development is the most important. The construction of environmental quality needs to be projected from the perspective of long-term strategy, including soil, air and water quality.

Second, the ecological civilization construction in some cities differs greatly from economic development. Therefore, it is imperative to promote coordinated development of economic construction, such as optimizing industrial structure and income distribution. The most important concern for economic quality is green economy construction. For example, the primary industry is supposed to attach importance to green agriculture, while the secondary industry should strengthen the pollution control and pollution monitoring. In addition, the tertiary industry is supposed to increase the strategic emerging industries.

Third, it is necessary to balance the level of ecological civilization among different areas and achieve unified development. The establishment of ecological civilization system has put forward requirements for the development of the cities. The ecological civilization is the development goal of the whole society, rather than individual region or city. Therefore, only by guaranteeing the unified development of regional ecological civilization development, can the country achieve the goal of ecological civilization construction.

Fourth, in order to protect the sustainable development of resources, it is supposed to improve the efficiency of resource utilization. Resource utilization efficiency includes land resource use efficiency, water use efficiency, forest resource use efficiency, etc. Utilizing renewable resources is supposed to ensure the renewability of the resource. When utilizing non-renewable resources, it is supposed to guarantee the maximum benefit ratio of resource utilization.

Fifth, the government is supposed to pay attention to the public’s satisfaction with the environment. The most important of ecological civilization development is to establish a good public living environment, including the natural environment and the human environment. Therefore, it is imperative to attach importance to the public’s attitude towards the environment.

## Figures and Tables

**Table 1:** Kaiser–Meyer–Olkin (KMO) and Bartlett sphericity test.

Inspection Content	Value
Kaiser–Meyer–Olkin	0.601
Bartlett $\chi^{2}$	2219.408
df	666
Sig.	0.000

**Table 2:** Variance interpretation table.

Component	Initial Eigenvalue			Extraction Sums of Squared Loadings		
	Total	% of Variance	% of Cumulative	Total	% of Variance	% of Cumulative
1	6.907	16.847	16.847	6.907	16.847	16.847
2	6.519	15.900	32.747	6.519	15.900	32.747
3	4.884	11.913	44.660	4.884	11.913	44.660
4	4.046	9.869	54.529	4.046	9.869	54.529
5	3.839	9.362	63.891	3.839	9.362	63.891
6	3.277	7.992	71.882	3.277	7.992	71.882
7	2.912	7.103	78.986	2.912	7.103	78.986
8	2.560	6.245	85.231	2.560	6.245	85.231

**Table 3:** Urban ecological civilization score based on STFA.

City	2012	2013	2014	2015	2016
Nanjing	72.17	51.69	73.87	65.49	52.67
Suzhou	4.93	19.80	11.44	−5.23	−2.65
Xuzhou	26.69	26.61	37.83	34.95	32.78
Wuxi	29.82	38.60	28.95	24.00	8.94
Zhenjiang	10.73	5.43	−9.42	0.43	−29.44
Taizhou	−3.21	−3.54	5.45	−11.75	−13.89
Suqian	28.80	9.96	28.21	13.44	18.21
Huai’an	20.37	17.42	−1.45	−3.96	1.28
Yancheng	−69.82	−76.97	−64.32	−55.13	−60.77
Lianyungang	−76.48	−32.56	−58.17	−79.62	−84.96
Yangzhou	−28.14	−28.88	−2.01	−0.44	−15.16
Nantong	−6.65	−15.04	−14.86	−31.08	−49.84
Changzhou	26.93	31.79	35.58	19.21	20.96

**Table 4:** Components and indexes for the ecological civilization construction.

Component	Indicators
fac1 Economic quality	Per capita disposable income of residents
	R&D expenditure/GDP
	Strategic emerging industries value/GDP
	Per capita GDP growth rate
	Tertiary industry added value/GDP
	Total energy consumption
	Total water consumption
fac2 Natural protection	Forest stock
	Forest cover rate
	Grassland comprehensive vegetation coverage
	Comprehensive utilization of crop straw
	Wetland protection rate
fac3 Cultivated land quality	Fertilizer use per unit cultivated land
	Pesticide use per unit cultivated land
fac4 Environmental Quality	Environmental pollution control investment/GDP
	Public satisfaction with the ecological environment
	Effective utilization coefficient of irrigation water
	Cultivated land holdings
fac5 City quality	Water consumption reduction per unit GDP
	Greenland rate in urban built-up areas
	Industry added value/water consumption reduction rate
	Green travel (public transport passenger traffic/population)
	Comprehensive utilization rate of general industrial solid waste
	Hazardous waste disposal utilization rate
	Harmless treatment rate of domestic garbage
fac6 Pollution control	PM_2.5_ emission reduction
	NO_X_ emission reduction
	SO_2_ emission reduction
	Chemical oxygen demand emission reduction
	CO_2_ emission reduction per unit GDP
	Good air quality rate
	Ammonia nitrogen e emission reduction
fac7 Rural construction	Rural clean toilet penetration rate
	Rural tap water penetration rate
fac8 Water quality	Surface water better than III type rate
	Surface water less than V type rate
	Energy consumption reduction per unit GDP

**Table 5:** Urban ecological civilization score based on Incidence Matrix.

	2012	2013	2014	2015	2016
Nanjing	12.12	22.91	13.89	18.37	25.35
Suzhou	−1.13	12.21	11.01	−3.38	6.95
Xuzhou	21.72	24.93	1.94	26.07	−1.90
Wuxi	−24.13	−18.46	−18.57	−14.76	−6.50
Zhenjiang	−16.75	−11.35	−5.68	7.00	−8.96
Taizhou	2.35	−4.15	28.80	−8.93	12.09
Suqian	0.14	−21.80	3.68	12.68	6.19
Huai’an	14.67	28.02	17.91	24.48	20.07
Yancheng	−4.51	−26.15	−32.24	−15.20	−10.31
Lianyungang	−12.37	−25.15	−11.27	−27.86	−6.80
Yangzhou	−3.24	7.33	12.75	5.15	7.70
Nantong	−11.61	5.83	−2.59	−4.82	−12.16
Changzhou	22.73	31.65	−9.63	−8.80	22.88

**Table 6:** Range of ratings through standard deviation method.

Rating	[0, V − B)	[V − B, V)	[V, V + B)	[V + B, 1]
	Low	Medium	High	Highest
Ecological Civilization Development	[0,0.27)	[0.27,0.51)	[0.51,0.79)	[0.79,1]
fac1	[0,0.13)	[0.13,0.42)	[0.42,0.71)	[0.71,1]
fac2	[0,0.05)	[0.05,0.32)	[0.32,0.58)	[0.58,1]
fac3	[0,0.18)	[0.18,0.50)	[0.50,0.82)	[0.82,1]
fac4	[0,0.02)	[0.02,0.26)	[0.26,0.49)	[0.49,1]
fac5	[0,0.29)	[0.29,0.52)	[0.52,0.76)	[0.76,1]
fac6	[0,0.28)	[0.28,0.47)	[0.47,0.65)	[0.65,1]
fac7	[0,0.34)	[0.34,0.52)	[0.52,0.70)	[0.70,1]
fac8	[0,0.17)	[0.17,0.42)	[0.42,0.67)	[0.67,1]
